# Ultrasound-guided SoracteLite™ transperineal laser ablation (TPLA) of the prostate for the treatment of symptomatic benign prostatic hyperplasia (BPH): a prospective single-center experience

**DOI:** 10.1007/s00345-023-04322-1

**Published:** 2023-02-28

**Authors:** Antonino Laganà, Giovanni Di Lascio, Aldo Di Blasi, Leslie Claire Licari, Antonio Tufano, Rocco Simone Flammia, Andrea De Carolis

**Affiliations:** 1 Ospedale San Giovanni Evangelista, Tivoli, Rome, Italy; 2 Policlinico Umberto I, Rome, Italy

**Keywords:** Transperineal laser ablation, Benign prostatic hyperplasia, Lower urinary tract symptoms, Laser therapy

## Abstract

**Purpose:**

To evaluate the efficacy and safety of ultrasound-guided transperineal laser ablation (TPLA) in patients with symptomatic BPH.

**Materials and methods:**

From January 2020 to January 2022, 63 prospectively enrolled patients underwent TPLA with a 1064-nm continuous-wave diode laser (EchoLaser, Elesta SpA). Primary endpoints were the change in IPSS, QoL, *Q*_max_, PVR and prostate volume at 3 and 12 months.

**Results:**

At 3 months, IPSS improved from 20.8 ± 7.4 to 11.0 ± 6.6 (*p* < 0.001), QoL from 4.7 ± 1.4 to 1.5 ± 1.2 (*p* < 0.001) and *Q*_max_ from 8.6 ± 3.5 mL/s to 13.2 ± 5.7 mL/s (*p* = 0.083). PVR decreased from 124.8 ± 115.4 mL to 43.6 ± 53.6 mL (*p* < 0.001), and prostate volume decreased from 63.6 ± 29.7 mL to 45.6 ± 21.8 mL (*p* = 0.003). At 12 months, IPSS improved from 20.8 ± 7.4 to 8.4 ± 5.9 (*p* < 0.001), QoL from 4.7 ± 1.4 to 1.2 ± 0.8 (*p* < 0.001), and *Q*_max_ from 8.6 ± 3.5 mL/s to 16.2 ± 4.3 mL/s (*p* = 0.014). PVR decreased from 124.8 ± 115.4 mL to 40.6 ± 53.6 mL (*p* = 0.003), and prostate volume decreased from 63.6 ± 29.7 mL to 42.8 ± 14.2 mL (*p* = 0.071). Transient complications consisted of two patients with prostatic abscess (Clavien-Dindo grade IIIa) and one patient with orchitis (Clavien-Dindo grade II).

**Conclusions:**

TPLA for symptomatic BPH provides clinical benefits at 3 and 12 months, and the treatment is well tolerated.

## Introduction

Benign prostatic hyperplasia (BPH) is caused by the proliferation of glandular epithelial tissue, smooth muscle, and connective tissue within the prostatic transition zone [1]. BPH is highly prevalent in the aging male population, with BPH and associated lower urinary tract symptoms (LUTS) such as urgency, difficulty initiating urination, incomplete bladder emptying, and decreased force of stream, increasing with age. BPH affects 70% of US men aged 60–69 years and 80% of those aged ≥ 70 years [2]. In the Rancho Bernardo study, a prospective, community-based study of aging in Southern California, 56% of men aged 50–79 years, 70% of men aged 80–89 years, and 90% of men aged ≥ 90 years reported LUTS [3], a trend that has been observed by other investigators [4]. BPH with LUTS is associated with a large burden of disease with a reduced quality of life (QoL) for patients [4, 5] and their partners [6, 7].

Guideline-mandated treatment approaches include watchful waiting, medical management, and surgical intervention [1, 8]. The most common medications are alpha-blockers, 5-alpha reductase inhibitors (5-ARIs) (either alone, or in combination with an alpha-blocker), and the phosphodiesterase 5 inhibitor, tadalafil [1, 8]. Transurethral resection of the prostate (TURP) remains the gold-standard surgical intervention in selected patients, but it is associated with peri- and post-operative morbidity, and long-term complications such as ejaculatory dysfunction, urethral strictures, incontinence, and bleeding.

Given the limitations of surgery for BPH, efforts have been made to develop minimally invasive treatment approaches for patients needing treatment beyond medication, with the intention of achieving good efficacy while minimizing complications. One such approach is ultrasound-guided SoracteLite™ transperineal laser ablation (TPLA), for which an emerging literature has appeared in the last few years [9–15]. Here, we report the results of 12 months of follow-up in a prospective, single-center cohort of 63 patients who underwent SoracteLite™ TPLA for symptomatic BPH.

## Materials and methods

This study was approved by the local ethics committee. Informed consent was obtained from all individual participants included in the study. Inclusion criteria were: (1) patients with BPH with several comorbidities; (2) patients with a desire to spare anterograde ejaculation; (3) patients intolerant of or poorly compliant to medical therapy, with no indication for surgery. Exclusion criteria were: (1) acute and chronic prostatitis; (2) prior prostatic abscess; (3) prostate volume > 85 mL; and (4) all patients with PSA > 4.0 ng/mL without a negative MRI scan or negative biopsy for prostate cancer.

**Fig. 1:**
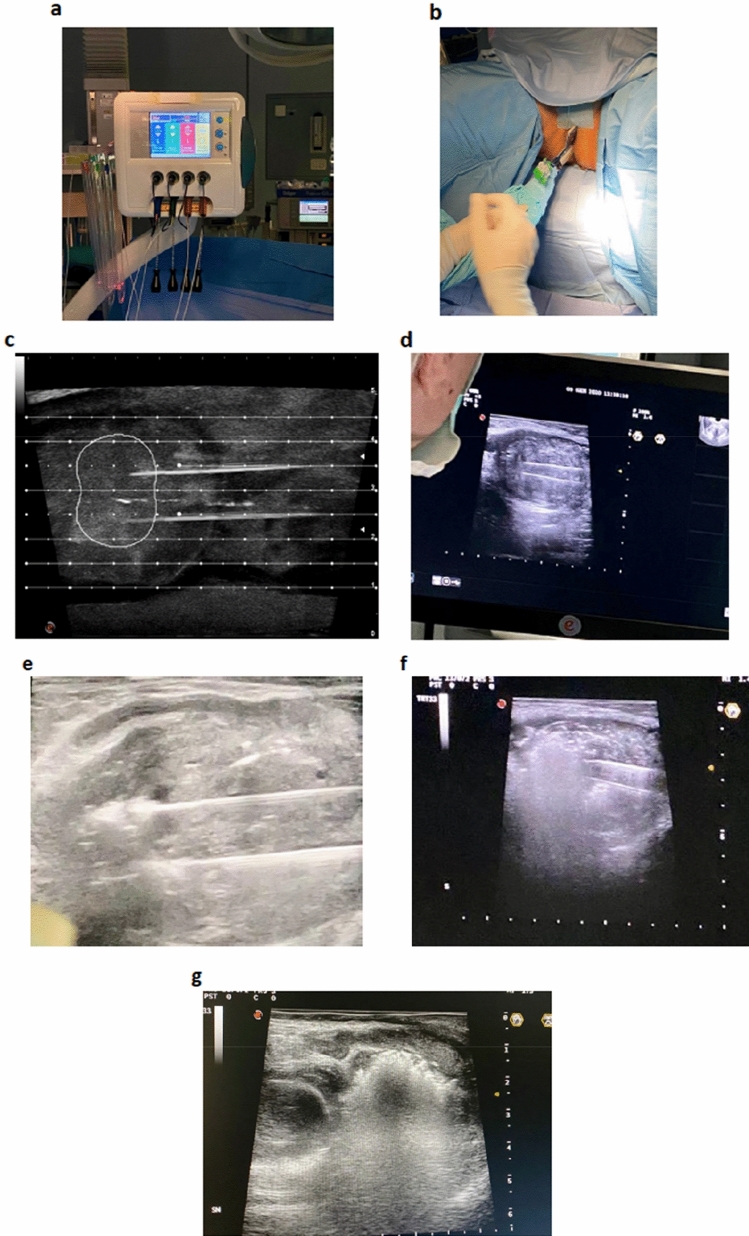
Illustration of the laser ablation procedure. **a** Power setting of each fiber of the multisource EchoLaser device with four independent channels; **b**, **c** To ease the insertion of needles and fibers, the transrectal US biplanar probe is combined with a multi-channel needle applicator, with a dedicated planning software displaying trajectories and safety distance area to help doctors to safely position applicators; **d** Longitudinal US image shows introducer needles positioned in the prostatic tissue; **e** Longitudinal US image shows hyperechoic effect in prostate tissue resulting from vapor bubble formation during laser energy delivery; **f** Longitudinal US image shows the second illumination of energy delivery after pull-back maneuver; **g** Longitudinal US image shows hyperechogenicity of the target area completely covered by the gas at the end of the treatment

Between January 2020 and January 2022, a total of 63 consecutive patients underwent SoracteLite™ TPLA at Ospedale San Giovanni Evangelista, Tivoli, Rome, Italy. In summary, SoracteLite™ TPLA procedure involves the coagulative necrosis of prostate tissue; this is achieved by laser illumination delivered by up to two laser fibers per prostatic lobe that are inserted transperineally under US guidance (Fig. 1). This heats the prostatic tissue and causes irreversible necrosis damage ‘in situ’ with no need to remove the tissue.

Procedures were performed by two surgeons (AL and A De C). SoracteLite™ TPLA was performed in the OR with the patient in the lithotomy position. Each treatment was performed with the patient under conscious sedation and with local anesthesia of the perineum followed by an US-guided periprostatic block. A three-way 18-F Foley catheter was inserted to permit continued refreshing water flux during the treatment. All patients received antibiotic prophylaxis (2 g cephazolin administered intravenously, with the infusion started before the start of the procedure). Patients were treated using EchoLaser system (Elesta SpA, Calenzano, Florence, Italy), a 1064-nm continuous-wave multi-source diode laser, in conjunction with a MyLab Eight Ultrasound device (Esaote, Genoa, Italy). Dedicated planning software in the US device was used to optimize applicator positioning within the prostate volume (Fig. 1c), taking into account safety distances to maintain from urethra, prostatic capsule and bladder neck. After the planning phase, either one or two 21G introducer needles were inserted per lobe, according to prostate dimensions and shape. The needles were inserted parallel to the prostatic capsule and urethra under US guidance using a transperineal approach (Fig. 1b, c). Then, a 300-μm optical laser fiber was introduced into each needle, with 10 mm protruding from the needle tip. Fiber tips were placed at a security distance of at least 15 mm from the bladder neck and 8–10 mm from the prostatic capsule and urethra. Ablation was performed at a fixed power of 3 W, with an energy delivery of 1800 J per fiber and illumination (Fig. 1a). Where necessary, a pull-back maneuver and re-illumination was performed (Fig. 1f). When two fibers were placed in the same lobe, a distance of 10 mm was maintained between them. Patients were discharged the same day as the SoracteLite™ TPLA procedure, with the urinary catheter in place.

At baseline, 3 months and 12 months, patients were asked to complete the International Prostate Symptom Score [IPSS] consisting of seven symptom questions and one question on QoL [16], and non-invasive urodynamics data (*Q*_max_, PVR) were collected. Prostate volume was also measured by transrectal ultrasonography at these three timepoints.

Primary endpoints were the IPSS, QoL, *Q*_max_, PVR and prostate volume at 3 and 12 months. Major complications, minor complications, and discontinuation of BPH drug therapy were recorded as secondary endpoints. Statistical analyses were performed using IBM SPSS statistics 27 (IBM SPSS, IBM Corp., Armonk, NY, USA). Values for quantitative variables are expressed mean and SD, or as median and range. Comparisons were performed using a Friedman test, with a *p*-value < 0.05 deemed to be statistically significant.

## Results

A total of 63 patients aged 72.3 ± 10.0 years with symptomatic BPH underwent TPLA. Table 1 summarizes the preoperative characteristics of the patients and the periprocedural data. All patients completed the 12-month follow-up.

**Table 1:** Patient characteristics and perioperative data

Variable	
*Patient characteristics*	
Age (years); mean ± SD	72.3 ± 10.0
BMI (kg/m^2^); mean ± SD	30.2 ± 7.1
CCI; median (range)	3 (2–5)
Patients with indwelling urinary catheter; *n* (%)	10 (15.9%)
Alpha-blocker therapy; *n* (%)	27 (42.9%)
5-ARI therapy; *n* (%)	6 (9.5%)
Prostate volume (mL); mean ± SD	63.6 ± 29.7
Preoperative PSA (ng/mL); mean ± SD	4.82 ± 1.8
*Perioperative data*	
Total energy delivered (J); mean ± SD	8,261.6 ± 3,280.1
Total procedure time (min); mean ± SD	48.8 ± 14.3
Ablation time (min); mean ± SD	13.0 ± 1.95
Number of fibers per lobe; mean ± SD	1.82 ± 0.4
Time without pull-back (min); mean ± SD	10 ± 2.1
Catheterization time (days); mean ± SD	
Overall	14.9 ± 7.5
Patients without indwelling catheter prior to TPLA	12.8 ± 4.9
Patients with indwelling catheter prior to TPLA	19.8 ± 9.8

*5-ARI* 5-alpha reductase inhibitor, *BMI* body mass index, *CCI* Charlson Comorbidity Index, *PSA* prostate-specific antigen, *SD* standard deviation, *TPLA* transperineal laser ablation

**Fig. 2:**
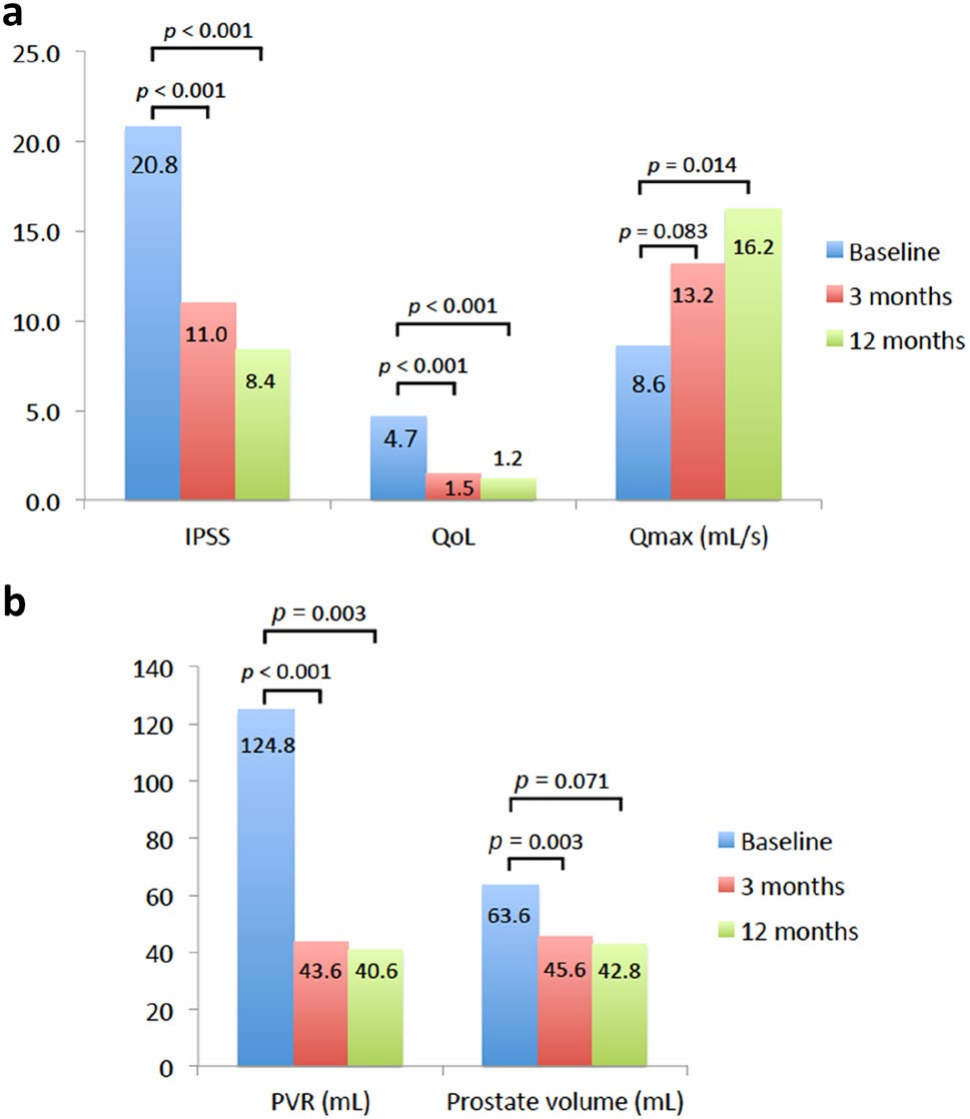
Clinical outcomes at 3 months and 12 months. **a** IPSS, QoL and *Q*_max_; **b** PVR and prostate volume. *IPSS* International Prostate Symptom Score, *QoL* quality of life, *Q*_*max*_ maximum urinary flow rate, *PVR* post-void residual

At 3-month follow-up, IPSS was significantly improved from 20.8 ± 7.4 to 11.0 ± 6.6 (*p* < 0.001), QoL significantly improved from 4.7 ± 1.4 to 1.5 ± 1.2 (*p* < 0.001) and *Q*_max_ increased numerically from 8.6 ± 3.5 mL/s to 13.2 ± 5.7 mL/s (*p* = 0.083) (Fig. 2a). PVR was significantly reduced from 124.8 ± 115.4 mL to 43.6 ± 53.6 mL (*p* < 0.001), and prostate volume significantly decreased from 63.6 ± 29.7 mL to 45.6 ± 21.8 mL (*p* = 0.003) (Fig. 2b). Of the 27 patients who were receiving alpha-blockers at baseline, 15 (55.6%) discontinued this therapy at 3-month follow-up. Of the six patients who were taking a 5-ARI pre-operatively, three (50%) discontinued this therapy.

At 12-month follow-up, IPSS was significantly improved from 20.8 ± 7.4 to 8.4 ± 5.9 (*p* < 0.001), QoL from 4.7 ± 1.4 to 1.2 ± 0.8 (*p* < 0.001), and *Q*_max_ significantly increased from 8.6 ± 3.5 mL/s to 16.2 ± 4.3 mL/s (*p* = 0.014) (Fig. 2a). PVR was significantly reduced from 124.8 ± 115.4 mL to 40.6 ± 53.6 mL (*p* = 0.003), and prostate volume decreased from 63.6 ± 29.7 mL to 42.8 ± 14.2 mL (*p* = 0.071) (Fig. 2b). At 12-month follow-up, all patients had discontinued medical therapy.

At 12-month follow-up, the mean PSA concentration was reduced to 2.89 ± 1.2 ng/mL from a pre-operative value of 4.82 ± 1.8 ng/mL.

In the subset of 10 patients who had an indwelling urinary catheter at baseline, all patients had their catheters removed at a mean of 19.7 ± 9.8 days after the procedure. At 12-month follow-up, mean prostate volume was reduced from 71.5 ± 17.3 mL at baseline to 48.5 ± 11.8 mL, IPSS was 14.1 ± 4.9, PVR was 37.5 ± 33.1 mL, and *Q*_max_ was 12.4 ± 5.2 mL/s.

Only transient complications occurred in this patient series. These complications consisted of two cases of prostatic abscess (Clavien–Dindo grade IIIa; 3.2% of patients) that were successfully drained, and one case of orchitis (Clavien-Dindo grade II; 1.6%) that was successfully treated with antibiotics.

## Discussion

TPLA for the treatment of BPH is a relatively new, micro-invasive approach that facilitates access to the prostate without the need for urethral instrumentation and the associated risk of adverse events. In addition, the procedure does not require the patient to undergo general anesthesia or to stay overnight in the hospital.

In the present study, statistically significant improvements at 3 months post-TPLA were observed for four of the five primary endpoints (IPSS, QoL, PVR and prostate volume), with a numerical improvement observed for the fifth (*Q*_max_). Although mean *Q*_max_ at 3 months was increased from 8.6 ± 3.5 mL/s to 13.2 ± 5.7 mL/s, the lack of statistical significance may reflect the high variability in the measurement of *Q*_max_ at this initial timepoint, particularly since prostate volume was significantly reduced at 3 months, and the increase in *Q*_max_ was statistically significant at 12 months. Furthermore, there was a relative improvement for each of the primary endpoints at 12 months vs 3 months. The latter observation most likely reflects the mechanism underlying laser ablation, i.e., coagulative tissue necrosis, with consequent removal of the necrotic tissue over time via the activity of macrophages [17].

Our study included a subset of 10 patients who had an indwelling urinary catheter before being treated with TPLA. In these patients, catheter removal was achieved at a mean of 19.8 days after the procedure, indicating the usefulness of TPLA in these challenging BPH patients. In this study, a catheter was placed in each patient treated, irrespective of whether he was catheterized prior to the treatment. Although SoracteLite TPLA has the potential to avoid or reduce the use of a urinary catheter in BPH patients [18], this was not an objective of the present study and should eventually be evaluated in a specifically designed new study.

To our knowledge, there have been seven previous studies on the use of SoracteLite™ TPLA for BPH [9–15], of which five have enrolled patients prospectively [9–12, 14]. In an initial prospective feasibility study, Patelli et al. [9] treated 18 patients with TPLA and analyzed clinical outcomes at 3 months, reporting significant improvements in IPSS, PVR, *Q*_max_, QoL and prostate volume compared with pretreatment values. Apart from our study, the largest prospective study with systematic follow-up at 12 months was reported by Manenti et al. [12], who treated 44 patients with TPLA and, in agreement with other studies, found improvements in IPSS, PVR, *Q*_max_, QoL and prostate volume. Using MRI, these investigators reported progressive reductions in volume of the necrotic area over 12 months of follow-up, consistent with the temporal evolution of clinical outcomes seen in the same study, in our study, and in the study by Frego et al. [11].

In our patient series, SoracteLite™ TPLA was well tolerated, with only three transient complications observed, i.e., prostatic abscess in two patients (Clavien–Dindo grade IIIa, 3.2% of patients) and one case of orchitis (Clavien–Dindo grade II, 1.6% of patients). Single cases of prostatic abscess or orchitis have been reported in three other studies [10, 12, 13], and so the current evidence suggests that these complications of TPLA occur only rarely and are transient (prostatic abscess: 0.6–4.8%; orchitis: 0.6–1.6% depending on the study). Overall, the published evidence indicates that the use of SoracteLite™ TPLA in BPH patients provides a favorable safety profile [9–15].

In addition to the micro-invasive SoracteLite™ TPLA procedure, a range of minimally invasive techniques is available for the treatment of BPH. Holmium Laser Enucleation of the Prostate (HoLEP) uses a contact optical fiber (end-firing fiber) that vaporizes and separates prostate tissue, with tissue fragments removed from the bladder by morcellation [19]. Rezūm water vapor thermal therapy uses radiofrequency to create thermal energy in the form of water vapor [20]. Aquabeam^®^ relies on image-guided, robot-assisted transurethral saline waterjet ablation (aquablation) of the prostate [21]. UroLift is another transurethral technique that uses a non-thermal approach to treat BPH, with deployed urinary stents acting to lift and hold the enlarged prostate tissue so that it no longer blocks the urethra [22].

While there is evidence for the efficacy of these techniques across a range of functional outcomes such as IPSS, QoL, and *Q*_max_, their reliance on a transurethral approach is associated with certain complications. HoLEP is associated with retrograde ejaculation in 75% of men undergoing full prostate enucleation, impotence in approximately 14%, and temporary incontinence in 10–15% [19]. The most common complications of Rezūm are dysuria in 16.9% of patients, hematuria (11.8%), and urinary frequency and urgency (5.9%) [23]. With Aquabeam, retrograde ejaculation has been reported in up to 20% of patients [8, 24, 25], bleeding requiring transfusion in up to 7.9% of patients [25], and urinary infection in up to 7.0% [24]. Complications of the UroLift procedure include hematuria in up to 63% of patients [8], urinary infection (2.9–11% of patients) [8], pelvic pain (5–17.9%) [8], and protrusion of the UroLift implants into the bladder in 8% of patients after 5 years [26]. These data highlight the safety advantages of the transperineal approach that in combination with very small applicators and mechanism of action allow TPLA to be an organ-sparing technique.

Certain strengths and limitations of this study should be mentioned. Patients were recruited prospectively, and our study is the largest published prospective study to report clinical outcomes at 12 months post-TPLA. However, our study lacked a control group and so does not enable a direct comparison with TURP—the gold standard—or with other minimally-invasive techniques. An ongoing multicenter, randomized, controlled trial that is comparing the efficacy and safety of TPLA and TURP [27] in men with BPH is expected to provide important information in this regard. The present study did not directly address cost-effectiveness. However, it is reasonable to predict that TPLA would compare favorably to conventional surgery on cost, because the procedure can be performed on an outpatient basis.

## Conclusions

In conclusion, TPLA represents an effective and safe treatment for symptomatic BPH, providing clinically significant benefits on prostate volume, bladder function and QoL at 3 months and 12 months.

## Data Availability

All data generated or analysed during this study are included in this published article.
